# Association of DGF and Early Readmissions on Outcomes Following Kidney Transplantation

**DOI:** 10.3389/ti.2022.10849

**Published:** 2022-12-23

**Authors:** Caroline C. Jadlowiec, Peter Frasco, Elizabeth Macdonough, Josiah Wagler, Devika Das, Pooja Budhiraja, Amit K. Mathur, Nitin Katariya, Kunam Reddy, Hasan Khamash, Raymond Heilman

**Affiliations:** ^1^ Division of Transplant Surgery, Department of Surgery, Mayo Clinic, Phoenix, AZ, United States; ^2^ Division of Anesthesiology, Mayo Clinic, Phoenix, AZ, United States; ^3^ Division of Gastroenterology and Hepatology, Mayo Clinic, Phoenix, AZ, United States; ^4^ Division of Internal Medicine, Mayo Clinic, Rochester, MN, United States; ^5^ Division of Nephrology, Mayo Clinic, Phoenix, AZ, United States

**Keywords:** outcomes, kidney transplant, delayed graft function, graft type, readmission

## Abstract

Concerns regarding outcomes and early resource utilization are potential deterrents to broader use of kidneys at risk for delayed graft function (DGF). We assessed outcomes specific to kidneys with DGF that required early readmission following transplant. Three groups were identified: 1) recipients with DGF not requiring readmission, 2) recipients with DGF having an isolated readmission, and 3) recipients with DGF requiring ≥2 readmissions. Most recipients either required a single readmission (26.8%, *n* = 247) or no readmission (56.1%, *n* = 517); 17.1% (*n* = 158), had ≥2 readmissions. Recipients requiring ≥2 readmissions were likely to be diabetic (53.8%, *p* = 0.04) and have longer dialysis vintage (*p* = 0.01). Duration of DGF was longer with increasing number of readmissions (*p* < 0.001). There were no differences in patient survival for those with DGF and 0, 1 and ≥2 readmissions (*p* = 0.13). Graft survival, however, was lower for those with ≥2 readmissions (*p* < 0.0001). This remained true when accounting for death-censored graft loss (*p* = 0.0012). Additional subgroup analysis was performed on mate kidneys with and without DGF and mate kidneys, both with DGF, with and without readmissions. For these subgroups, there were no differences in patient or graft survival. As a whole, patients with DGF have excellent outcomes, however, patients with DGF requiring ≥2 readmissions have lower graft survival. A better understanding of recipient variables contributing to multiple readmissions may allow for improvements in the utilization of DGF at-risk kidneys.

## Graphical Abstract

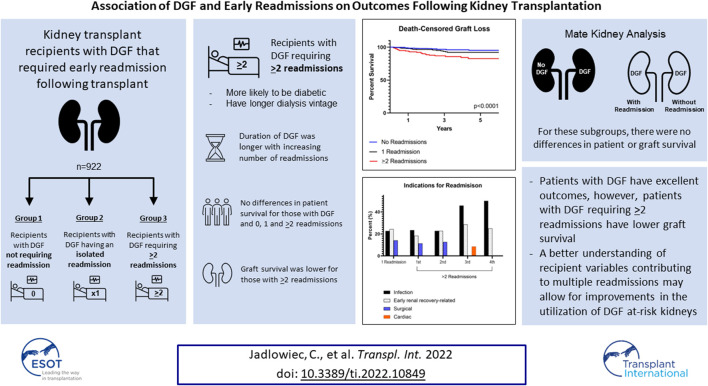

## Introduction

Delayed graft function (DGF) is a common post-transplant event. Although the incidence varies between transplant centers, it is known to occur at a higher rate with certain types of kidney allografts, such as those coming from high kidney donor profile index (KDPI) donors, acute kidney injury (AKI) donors and donation after circulatory death (DCD) donors (1-3). The clinical significance of DGF and its impact on outcomes remains debated, however outcomes-related concerns, in combination with increased need for early resource utilization, are perceived as deterrents to the broader use of kidneys at risk for DGF (1-8). These factors unfortunately predispose certain kidney allografts to underutilization and place them at a high risk for discard (8). Although donor-related factors contributing to DGF are well established, recipient-specific variables likely also play an important role in DGF, resource utilization, and transplant outcomes (9-10). Our center has gained experience in using DGF at-risk kidneys and managing post-transplant events in the outpatient setting (1-3). Given this background, we sought to assess variables and outcomes specific to kidneys with DGF that required early readmission following transplant.

## Methods

**FIGURE 1:**
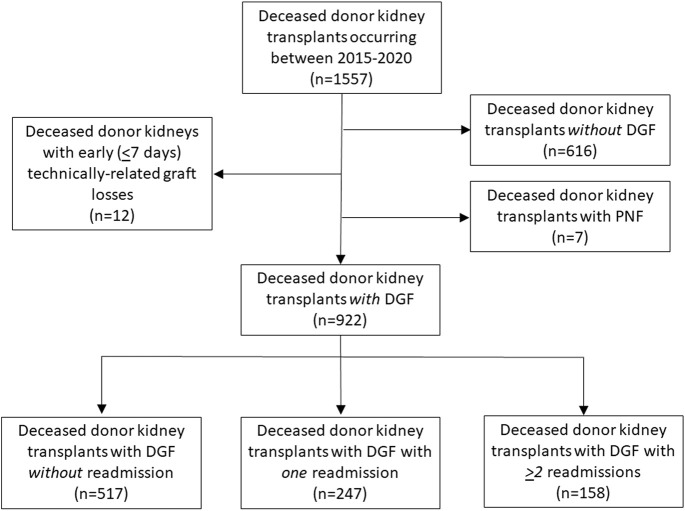
Study design flowchart

This is a retrospective review of patients with DGF who received deceased donor kidney transplants at Mayo Clinic Arizona from 2015 through 2020. Recipients with DGF were assessed based on their need for readmission. Three groups were identified: 1) recipients with DGF not requiring readmission, 2) recipients with DGF having a single isolated readmission, and 3) recipients with DGF requiring ≥2 readmissions. Living donor kidney transplants and multivisceral transplants were not included in this analysis. Recipients with early (<7 days post-transplant) technically related graft losses (*n* = 12) and with primary nonfunction (*n* = 7) were excluded as were recipients of deceased donor kidneys without DGF (*n* = 616) (Figure 1). The study was deemed exempt by the Institutional Review Board (IRB 20-000860).

DGF was defined as the need for dialysis within 7 days of kidney transplant. Acute kidney injury (AKI) donors were defined as those with Acute Kidney Injury Network (AKIN) stage 2-3 (2, increase in serum creatinine >twofold to threefold from baseline; 3, increase in serum creatinine >4.0 mg/dl or >threefold from baseline or requirement of renal replacement therapy) (1-3).

Data on readmissions was obtained using the electronic health record. The electronic health record was queried for the date of admission, date of transplant procedure, date of discharge and initial length of stay. Early readmission following kidney transplant was defined as occurring within 60 days of the index procedure. A readmission was defined as any hospital stay ≥24 h. The International Classification of Diseases (ICD) 10 codes (ICD 9 prior to October 2015) for the primary readmission discharge diagnosis were recorded. Readmissions related to early renal recovery include those attributed to volume status (overload or dehydration) and electrolyte management. Percentages of missing variables are noted in Supplementary Table S1.

Protocolized induction and maintenance immunosuppression was used for all kidney transplant recipients. Basiliximab induction was used for patients over 65 years of age; patients less than 65 years of age received depleting induction. Those who received basiliximab were continued on maintenance corticosteroids while steroid discontinuation occurred by post-transplant day five for those receiving depleting induction. Tacrolimus and mycophenolate mofetil were used for maintenance immunosuppression. Tacrolimus was started on post-transplant day 1-2 irrespective of DGF. Tacrolimus trough levels were maintained between 8–10 ng/ml for the first month post-transplant and between 6–8 ng/ml after 1 month. All reported rejections were biopsy-proven. Early acute cellular rejection (ACR) was defined as occurring within 6 months of transplant. Estimated GFR (eGFR) was calculated using the CKD-EPI formula. Six-minute walk distance was used to assess candidate suitability for transplant as previously described (11).

Recipients are typically discharged between post-transplant days 2 and 3 regardless of DGF (12). For those with DGF, dialysis occurred as an outpatient in a community-based dialysis unit. Need to discontinue dialysis was monitored in the outpatient setting with clinic visits and laboratory studies occurring 2-3 times per week. Parameters used to guide discontinuation of dialysis included serum laboratory studies, recipient weight and urine output volume.

### Outcomes

Primary outcomes were post-transplant hospital length of stay, early ACR, eGFR, and patient and allograft survival comparing recipients with DGF having 0, 1 and ≥2 post-transplant readmissions. Secondary outcomes were obtained through subgroup analyses on mate kidneys. Two subgroup analyses were completed: 1) mate kidney with and without DGF, and 2) mate kidneys both with DGF but with and without hospital readmissions. Primary outcomes were applied to the subgroup analyses.

### Statistical Methods

Chi-square analysis was used for categorical variables and t-tests were used for quantitative variables. Graft and patient survival were calculated by Kaplan-Meier survival analysis. We also used a Cox proportional hazard model to adjust for baseline differences in death-censored graft survival. A *p*-value of less than 0.05 was considered statistically significant. Descriptive statistics were reported as mean ± standard deviation, mean ± standard deviation and median, or median and interquartile range (IQR); categorical variables were reported as count and percent. Data was analyzed using GraphPad Prism 9.3.1 (2021 GraphPad Software, Inc.) and BlueSky (Version 7.40).

## Results

In total, there were 1557 kidney transplants during this time period. Of those, 59.2% (*n* = 922) had DGF. Of these 922 kidneys with DGF, 13.3% were high KDPI (*n* = 123), 29.7% (*n* = 275) were from DCD donors, and 48.4% (*n* = 446) were from AKI donors. Characteristics of recipients with DGF requiring 0, 1 and ≥2 readmissions are shown in Table 1. Most recipients either required an isolated (single) readmission (26.8%, *n* = 247) or no readmission (56.1%, *n* = 517); 17.1% (*n* = 158), had ≥2 readmissions. In general, recipients were similar in age (*p* = 0.11) and race (*p* = 0.65). Recipients for all groups were more likely to be male (*p* = 0.56) and be on dialysis at the time of transplant (*p* = 0.16). Recipients requiring ≥2 readmissions were more likely to be diabetic (53.8%, *p* = 0.04) and have longer dialysis vintage (median 3.4 years, *p* = 0.01). There were no differences observed between the three groups with regard to pre-transplant ejection fraction (*p* = 0.68), 6-minute walk distance (*p* = 0.07) and need for midodrine (*p* = 0.18).

**TABLE 1:** Characteristics of recipients and donors with DGF and 1, ≥2 or no readmissions.

	DGF No readmission (n = 517)	DGF Single readmission (n = 247)	DGF ≥2 readmissions (n = 158)	*p*-value
Recipient				
Age (years)	55.3 ± 12.8 (57.0)	57.1 ± 12.8 (60.0)	57.3 ± 12.7 (59.0)	0.11
Male	333 (64.4%)	160 (64.8%)	109 (69.0%)	0.56
Race				
White	214 (41.4%)	96 (38.9%)	71 (44.9%)	0.65
Black	74 (14.3%)	36 (14.6%)	20 (12.7%)	
Hispanic	126 (24.4%)	75 (30.4%)	41 (25.9%)	
American Indian/Alaska Native	58 (11.2%)	23 (9.3%)	12 (7.6%)	
Other	45 (8.7%)	17 (6.9%)	14 (8.9%)	
BMI (kg/m^2^)	28.8 ± 5.6 (28.3)	28.5 ± 5.3 (28.4)	28.7 ± 5.8 (28.4)	0.78
Diabetes	222 (42.9%)	121 (49.0%)	85 (53.8%)	0.04
Ejection Fraction	61.6 ± 6.8 (62.0)	61.1 ± 7.2 (62.0)	60.7 ± 6.7 (61.0)	0.68
EF <45%	15 (2.9%)	11 (4.5%)	5 (3.2%)	0.53
6-minute walk distance (m)	375.5 ± 73.8 (366.0)	333.9 ± 67.5 (344.5)	372.8 ± 49.4 (367.5)	0.07
Midodrine pre-transplant	6 (1.2%)	3 (1.2%)	5 (3.2%)	0.18
Preemptive	45 (8.7%)	13 (5.3%)	9 (5.7%)	0.16
Length of dialysis (years)	3.6 ± 2.6 (3.2)	4.2 ± 3.3 (3.6)	4.0 ± 2.9 (3.4)	0.01
Re-Transplant	41 (7.9%)	23 (9.3%)	17 (10.8%)	0.52
Donor				
Age (years)	40.3 ± 14.8 (39.0)	41.5 ± 15.3 (41.0)	39.9 ± 15.3 (38.0)	0.46
Male	326 (63.1%)	140 (56.7%)	93 (58.9%)	0.21
Height (cm)	170.2 ± 13.8 (172.0)	168.3 ± 14.5 (168.0)	169.0 ± 16.1 (170.0)	
KDPI (%)	52.6 ± 25.1 (51.0)	56.4 ± 23.6 (53.0)	53.1 ± 24.7 (53.0)	0.13
High KDPI	64 (12.4%)	41 (16.6%)	18 (11.4%)	0.20
DCD	147 (28.4%)	82 (33.2%)	46 (29.1%)	0.40
AKI	256 (49.5%)	116 (47.0%)	74 (46.8%)	0.73
Allocation				
Local	226 (43.7%)	89 (36.0%)	60 (38.0%)	0.15
Regional	115 (21.1%)	70 (28.3%)	41 (25.9%)	
National	147 (35.2%)	88 (35.6%)	57 (36.1%)	
Induction				
Alemtuzumab	320 (61.9%)	145 (58.7%)	87 (55.1%)	0.62
Basiliximab	161 (31.1%)	82 (33.2%)	58 (36.7%)	
Thymoglobulin	36 (7.0%)	20 (8.1%)	13 (8.2%)	
CIT (hours)	20.7 ± 6.2 (21.4)	21.0 ± 6.4 (21.4)	21.4 ± 6.4 (21.7)	0.49

Donor characteristics for the three groups are shown in Table 1. Overall, there were no differences noted. Donors were similar in age (*p* = 0.46) and more likely to be male (*p* = 0.21). The median KDPI score was 52.0% (*p* = 0.13); high KPDI (KDPI ≥85%) kidneys were equally distributed between the three groups (12.4% vs. 16.6% vs. 11.4%, *p* = 0.20). A similar distribution of AKI (49.5% vs. 47.0% vs. 46.8%, *p* = 0.73) and DCD (28.4% vs. 33.2% vs. 29.1%, *p* = 0.40) kidneys allografts was observed between the groups. Distribution of locally, regionally, and nationally allocated kidneys (*p* = 0.15) and cold ischemia time (CIT, median 21.4 h, *p* = 0.49) were also similar and did not vary between the three groups. Alemtuzumab was the most commonly used induction agent for all groups (*p* = 0.62).

Post-operative outcomes for recipients with DGF requiring 0, 1 and ≥2 readmissions are shown in Table 2. Duration of DGF increased along with number of readmissions. Median DGF duration was 9 days for those not requiring readmission, 10 days for those with an isolated readmission and 13 days for those requiring ≥2 readmissions (*p* < 0.001). Readmissions occurred later post-transplant for those recipients with one readmission compared to those with ≥2 readmissions (median 18 vs. 13 days, *p* < 0.001). Recipients with one readmission were also more likely to have had resolution of DGF prior to readmission compared to those with ≥2 readmissions (median 6 vs. 1 day(s), *p* < 0.001). Despite differences in duration of DGF, there were no differences in initial hospital length of stay (LOS) (median 3.0 days, *p* = 0.91) or early ACR events (*p* = 0.31). At 4 months post-transplant, there were no differences between the groups with regard to overall eGFR (*p* = 0.26), although the percentage of individuals with an eGFR <30 ml/min was higher for those requiring ≥2 readmissions (9.7% vs. 11.0% vs. 19.5%, *p* = 0.007). At 1- and 2-years post-transplant, there were no differences in overall eGFR (1-year, *p* = 0.11; 2-year, *p* = 0.86) or eGFR <30 ml/min (1-year, *p* = 0.08; 2-year, *p* = 0.88).

**TABLE 2:** Post-operative outcomes of recipients and donors with DGF and 1, ≥2 or no readmissions.

	DGF No readmission	DGF Single readmission	DGF ≥2 readmissions	*p*-value
LOS	3.0 (2.0, 3.0)	3.0 (2.0, 3.0)	3.0 (2.0, 4.0)	0.91
DGF days	10.5 ± 10.8 (9.0)	12.2 ± 9.8 (10.0)	16.5 ± 15.2 (13.0)	<0.001
Time to readmission (days)	—	18 (9, 30)	13 (7,22)	<0.001
ACR	21 (4.1%)	8 (3.2%)	10 (6.3%)	0.31
eGFR (ml/min)				
4 months	50.2 ± 15.9 (50.0)	49.2 ± 16.8 (50.0)	47.5 ± 18.5 (47.0)	0.26
eGFR <30 ml/min	45 (9.7%)	23 (11.0%)	26 (19.5%)	0.007
1 year	52.9 ± 17.3 (53.0)	53.7 ± 17.2 (55.3)	49.4 ± 19.7 (46.0)	0.11
eGFR <30 ml/min	36 (7.6%)	16 (8.4%)	16 (14.2%)	0.08
2 years	50.1 ± 17.7 (49.9)	51.3 ± 17.7 (51.0)	50.5 ± 19.9 (50.0)	0.86
eGFR <30 ml/min	26 (13.4%)	14 (11.7%)	8 (11.8%)	0.88

**FIGURE 2:**
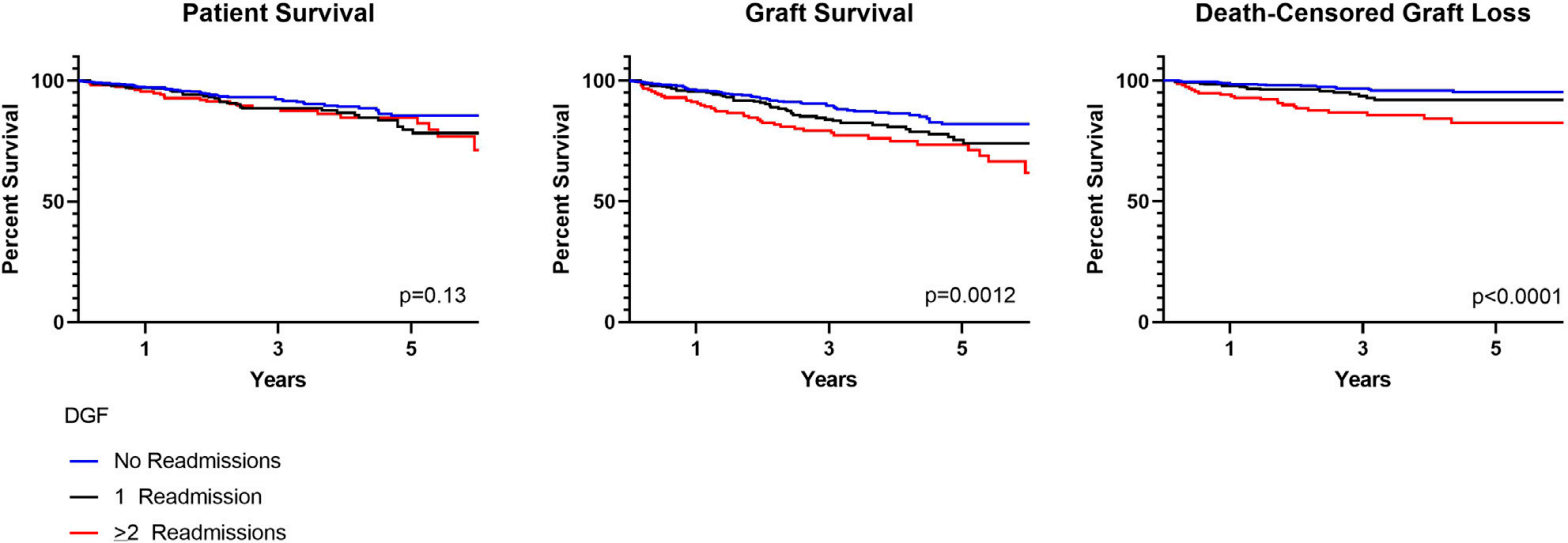
Patient and graft survival in kidneys with DGF and 0, 1, ≥2 readmissions.

There were no differences in patient survival for those with DGF and 0, 1 and >2 readmissions (*p* = 0.13). Graft survival, however, was lower for those with ≥2 readmissions (*p* = 0.0012). This remained true when accounting for death-censored graft loss (*p* < 0.0001) (Figure 2). At 1 year, patient survival was 97.3%, 97.2% and 95.6% for those with 0, 1 and ≥2 readmissions; kidney graft survival was 96.1%, 95.5%, and 91.1%. Median follow-up was 2.7 years (IQR 2.1–6.6) for recipients with 0 readmissions, 3.1 years (IQR 2.1–4.9) for recipients with one readmission and 3.1 years (IQR 2.0–5.0) for recipients with ≥2 readmissions. Cardiopulmonary events accounted for the most common cause of patient death occurring less than 1-year post-transplant in all groups. Death with function followed by infection accounted for the most common causes of graft loss occurring less than 1-year post-transplant. In a cox proportional hazards regression model accounting for presence of pre-transplant diabetes and dialysis duration, ≥2 readmissions was associated with an increased risk for death-censored graft loss (HR 3.1, 95% CI 1.8–5.3) (Supplementary Table S1).

### Causes for Post-Transplant Readmission

**FIGURE 3:**
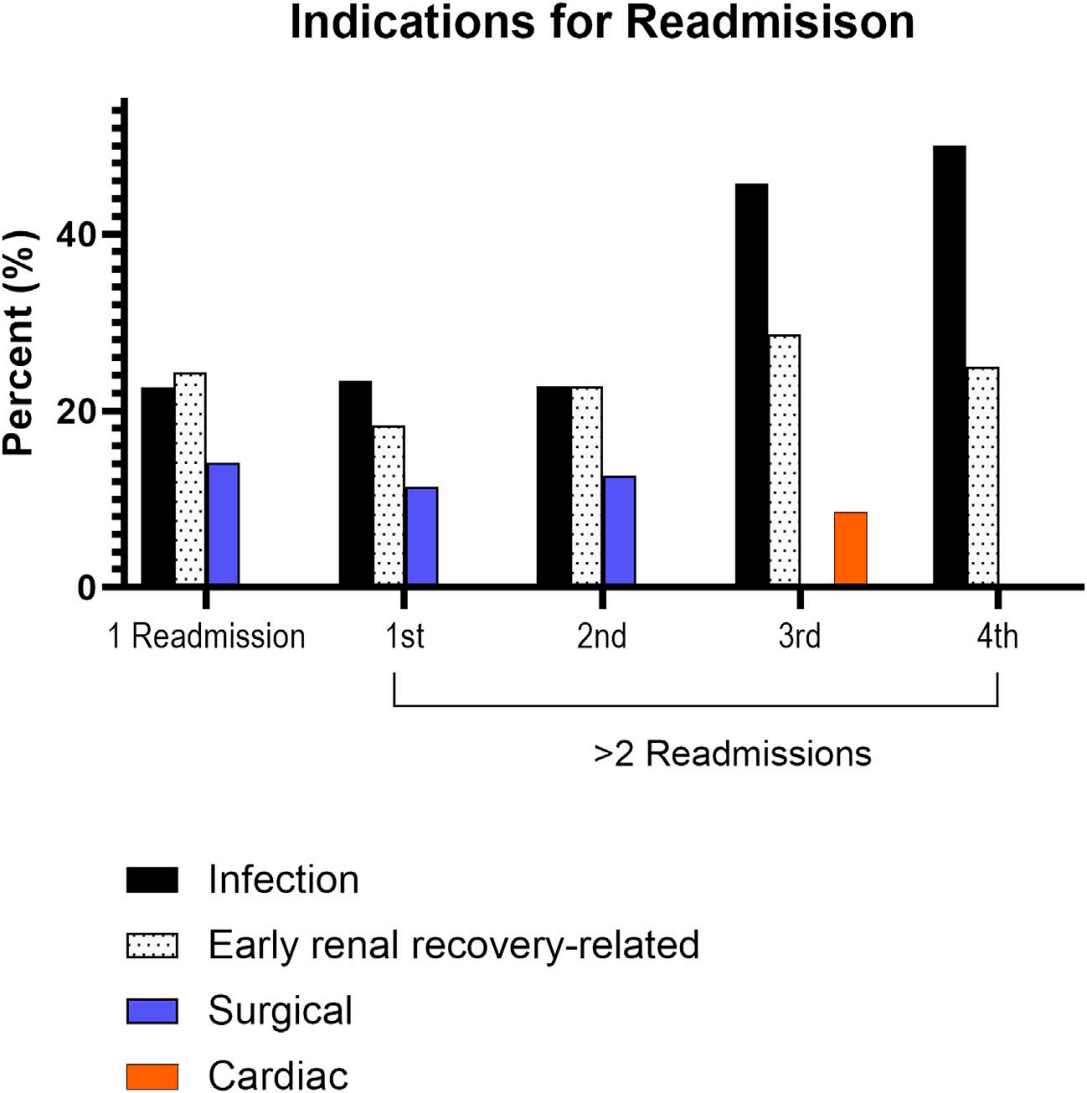
Leading causes for readmission. There were 35 patients with three readmissions, 12 patients with four readmissions, 2 patients with five readmissions, and 1 patient with six readmissions.

In assessing the initial index readmission for those with 1 and ≥2 readmissions, infection, early renal recovery related factors, and surgical complications were the most common indications observed (Figure 3). Subsequent readmissions for those with ≥2 readmissions are shown in Figure 3. Infection and factors related to early renal recovery remained the most common causes for readmission in subsequent readmissions.

### Subgroup Analysis on Mate Kidneys With and Without DGF

Of the 922 kidney transplants with DGF, 111 had mate kidneys without DGF. Patient characteristics for mate kidneys with and without DGF are shown in Table 3. Recipients of mate kidneys with DGF were more likely to be male (67.6% vs. 50.5%, *p* = 0.01) and less likely to be preemptive (12.6% vs. 50.5%, *p* < 0.0001). Other characteristics such as age (*p* = 0.11), race (*p* = 0.23), presence of diabetes (*p* = 0.08), and dialysis vintage (*p* = 0.94) were similar between the two groups.

**TABLE 3:** Recipient and donor characteristics of mate kidneys with and without DGF.

	Mate kidney A, with DGF (*n* = 111)	Mate kidney B, without DGF (*n* = 111)	*p*-value
Recipient			
Age (years)	56.0 ± 13.2 (58.0)	58.7 ± 12.0 (62.0)	0.11
Male	75 (67.6%)	56 (50.5%)	0.01
Race			
White	53 (47.7%)	63 (56.8%)	0.23
Black	16 (14.4%)	14 (12.%)	
Hispanic	21 (18.9%)	15 (13.5%)	
American Indian/Alaska Native	7 (6.3%)	12 (10.8%)	
Other	14 (12.6%)	7 (6.3%)	
BMI (kg/m^2^)	27.9 ± 5.4 (27.5)	27.8 ± 5.6 (27.6)	0.92
Diabetes	40 (36.0%)	28 (25.2%)	0.08
Preemptive	14 (12.6%)	56 (50.5%)	<0.0001
Length of dialysis	3.4 ± 2.4 (3.0)	3.4 ± 2.6 (3.1)	0.94
Re-transplant	13 (11.7%)	8 (7.2%)	0.25
Donor			
Age (years)	39.1 ± 15.4 (37.0)		—
Male	69 (62.2%)		—
Height (cm)	169.8 ± 12.5 (170.4)		—
KDPI (%)	49.1 ± 25.7 (49.0)		—
High KDPI	14 (12.6%)		—
DCD	32 (28.8%)		—
AKI	51 (46.0%)		—
Allocation			
Local	54 (48.6%)		—
Regional	26 (23.4%)		
National	31 (27.9%)		
Induction			
Alemtuzumab	66 (59.5%)	62 (55.9%)	0.27
Basiliximab	34 (30.6%)	43 (38.7%)	
Thymoglobulin	11 (9.9%)	6 (5.4%)	
CIT (hours)	20.4 ± 6.7 (20.5)	19.8 ± 7.0 (21.0)	0.48

Median donor age was 37 years and 62.2% of donors were male (Table 4). The median KDPI score was 49.0%; 12.6% of allografts were high KDPI, 28.8% came from DCD donors, and 46.0% came from AKI donors. Alemtuzumab remained the most common induction agent used for both groups (*p* = 0.27). There were no differences in CIT (*p* = 0.48).

**TABLE 4:** Post-operative outcomes for mate kidneys, with and without DGF.

	Mate kidney A, with DGF	Mate kidney B, without DGF	*p*-value
Length of stay (days)	3.0 (2.0, 3.0)	2.0 (2.0, 3.0)	0.002
DGF duration (days)	12.8 ± 11.7 (11.0)	—	—
ACR	4 (3.6%)	5 (4.5%)	0.73
Readmission	48 (43.2%)	33 (29.7%)	0.04
Number readmissions/patient	1.0 (1.0, 2.0)	1.0 (1.0, 1.0)	0.08
Number of readmissions			0.02
None	63 (56.8%)	78 (70.3%)	
One	28 (25.2%)	26 (23.4%)	
≥ Two	20 (18.0%)	7 (6.3%)	
eGFR (ml/min)			
4 months	47.4 ± 17.1 (47.2)	47.9 ± 13.8 (50.0)	0.80
eGFR <30 ml/min	17 (17.2%)	3 (3.0%)	0.0008
1 year	52.2 ± 18.5 (51.2)	52.4 ± 17.1 (53.0)	0.94
eGFR <30 ml/min	9 (9.7%)	5 (5.6%)	0.29
2 years	49.7 ± 18.3 (49.0)	51.0 ± 17.8 (51.0)	0.69
eGFR <30 ml/min	9 (16.7%)	2 (3.4%)	0.01

Post-operative outcomes are shown in Table 4. Hospital length of stay was longer in mate kidneys with DGF (median 3.0 vs. 2.0 days, *p* = 0.002) and the median duration of DGF was 11.0 days. Readmissions were more common for mate kidneys with DGF (43.2% vs. 29.7%, *p* = 0.04). Although the overall number of readmissions per recipient did not vary between those with and without DGF (median 1.0, *p* = 0.08), mate kidneys mate kidneys with DGF were more likely to have >2 readmissions (18.0% vs. 6.3%, *p* = 0.02). Early ACR events were uncommon and did not vary between the two groups (3.6% vs. 4.5%, *p* = 0.75). There were no differences in overall eGFR at 4-months (*p* = 0.80), 1-year (*p* = 0.94) and 2-year (*p* = 0.69) although eGFR <30 ml/min was more commonly observed in mate kidneys with DGF at 4 months (17.2% vs. 3.0%, *p* = 0.0008) and 2 years (16.7% vs. 3.4%, *p* = 0.01).

**FIGURE 4:**
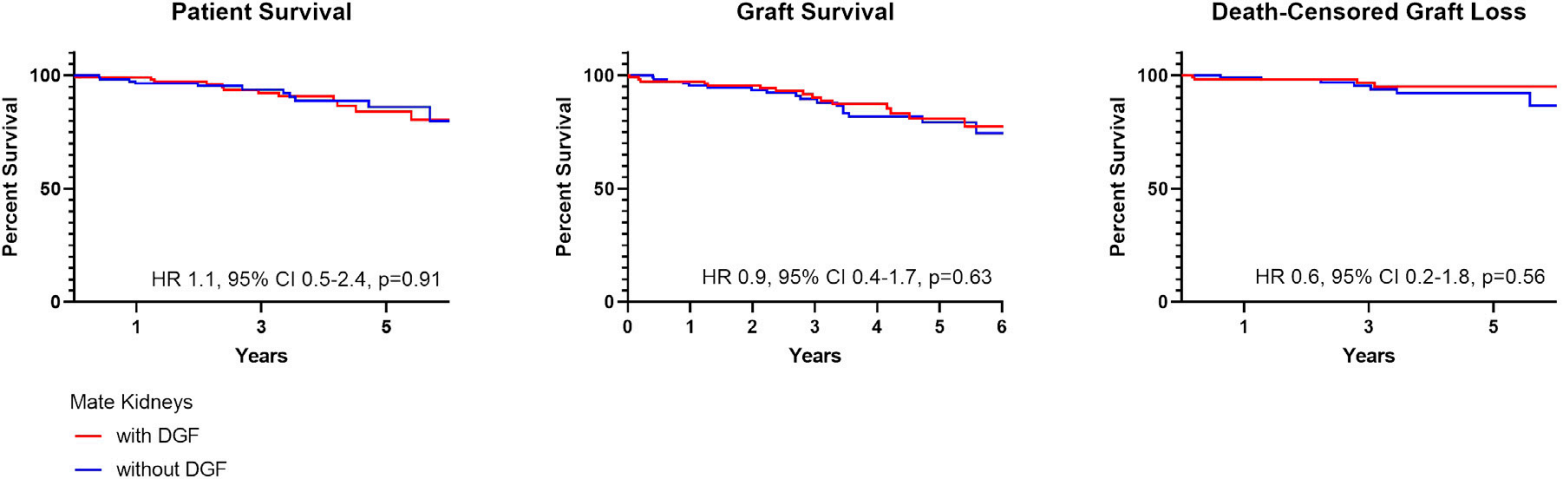
Patient and graft survival in mate kidneys, with and without DGF.

In comparing mate kidneys with and without DGF, there were no differences in patient (HR 1.1, 95% CI 0.5–2.4, *p* = 0.91) or graft survival (HR 0.9, 95% CI 0.4–1.7, *p* = 0.63) (Figure 4). This remained true when accounting for death-censored graft loss (HR 0.6, 95% CI 0.2–1.8, *p* = 0.56).

### Subgroup Analysis for Mate Kidneys With DGF With and Without Readmission

Of the 922 kidney transplants with DGF, there were 89 mate kidneys with and without readmission. Recipient characteristics of DGF mate kidneys with and without readmission are shown in Table 5. Recipient characteristics were overall similar between the two groups with no differences noted in age (*p* = 0.26), body mass index (BMI) (*p* = 0.89), and presence of diabetes (*p* = 0.29). Recipients requiring readmission were more likely to be male (75.3% vs. 57.3%, *p* = 0.01). A similar distribution of preemptive recipients was noted in both groups (2.3% vs. 6.7%, *p* = 0.15). There were no differences in dialysis vintage (*p* = 0.53).

**TABLE 5:** Recipient and donor characteristics of mate kidneys, both with DGF, with and without readmission.

	DGF, mate kidney A, readmission (*n* = 89)	DGF, mate kidney B, no readmission (*n* = 89)	*p*-value
Recipient			
Age (years)	55.8 ± 12.2 (55.0)	57.8 ± 11.6 (59.0)	0.26
Male	67 (75.3%)	51 (57.3%)	0.01
Race			
White	31 (34.8%)	38 (42.7%)	0.75
Black	15 (16.9%)	14 (15.7%)	
Hispanic	26 (29.2%)	22 (24.7%)	
Other	17 (19.1%)	15 (16.9%)	
BMI (kg/m^2^)	29.1 ± 5.5 (28.8)	29.2 ± 5.5 (29.2)	0.89
Diabetes	45 (50.6%)	38 (42.7%)	0.29
Preemptive	2 (2.3%)	6 (6.7%)	0.15
Length of dialysis	4.0 ± 2.7 (3.6)	3.7 ± 1.8 (3.6)	0.53
Re-transplant	6 (6.7%)	4 (4.5%)	0.52
Donor			
Age (years)	39.7 ± 14.0 (38.0)		—
Male	134 (64.1%)		—
Height (cm)	170.4 ± 13.2 (171.5)		—
KDPI (%)	53.0 ± 23.7 (51.0)		—
High KDPI	27 (12.9%)		—
DCD	62 (29.7%)		—
AKI	122 (58.4%)		—
Allocation			
Local	86 (41.1%)		—
Regional	47 (22.5%)		
National	76 (36.4%)		
Induction			
Alemtuzumab	56 (62.9%)	50 (56.2%)	0.27
Basiliximab	27 (83.1%)	36 (40.4%)	
Thymoglobulin	6 (6.7%)	3 (3.4%)	
CIT (hours)	21.3 ± 5.8 (21.3)	21.1 ± 6.0 (21.6)	0.88

The median donor age was 38 years and 64.1% of donors were male (Table 5). Median KDPI was 51.0%; 12.9% of kidneys were high KDPI, 29.7% were from DCD donors, and 58.4% came from AKI donors. There were no differences in induction with alemtuzumab being the most commonly used induction agent (*p* = 0.27). CIT was similar for both groups (*p* = 0.88).

**FIGURE 5:**
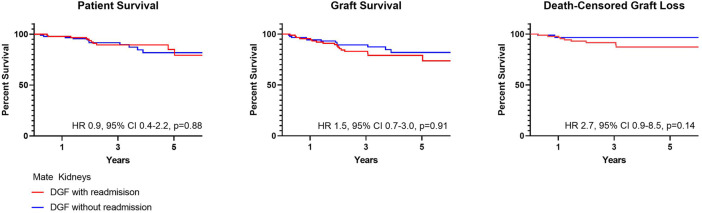
Patient and graft survival in mate kidneys, both with DGF, with and without readmissions.

Post-Transplant outcomes from DGF mate kidneys with and without readmission are shown in Table 6. There were no differences in DGF duration (*p* = 0.98), hospital length of stay (*p* = 0.96) and early ACR events (*p* > 0.99). There likewise were no differences in patient (HR 0.9, 95% CI 0.4–2.2, *p* = 0.88) or graft survival (HR 1.5, 95% CI 0.7–3.0, *p* = 0.91) (Figure 5).

**TABLE 6:** Post-operative outcomes of mate kidneys, both with DGF, with and without readmission.

	DGF, mate kidney A, readmission	DGF, mate kidney B, no readmission	*p*-value
Length of stay (days)	3.0 (2.0, 4.0)	3.0 (2.0, 4.0)	0.96
DGF duration (days)	12.8 ± 7.6 (12.0)	12.9 ± 12.9 (11.0)	0.98
ACR	6 (6.7%)	6 (6.7%)	>0.99
Number of readmissions			—
None	—	89 (100%)	
One	64 (71.9%)		
≥2 Two	25 (28.1%)		
eGFR (ml/min)			
4 months	50.2 ± 17.2 (50.0)	51.1 ± 15.9 (53.0)	0.73
eGFR <30 ml/min	7 (7.9%)	8 (9.0%)	0.79
1 year	52.6 ± 19.6 (56.4)	53.6 ± 16.7 (58.0)	0.76
eGFR <30 ml/min	8 (9.0%)	6 (6.7%)	0.58
2 years	52.7 ± 22.7 (50.8)	45.7 ± 16.6 (46.8)	0.16
eGFR <30 ml/min	6 (6.7%)	7 (7.9%)	0.77

## Discussion

Concerns related to outcomes, along with increased need for early resource utilization, such as dialysis and hospital readmissions, are believed to be deterrents to the broader use of kidneys at risk for DGF (1-8). Although certain types of kidney allografts are at increased risk for DGF, recipient-specific variables likely play an equally important role in DGF, resource utilization, and transplant outcomes (9-10). As such, the aim of this study was to assess variables and outcomes specific to kidneys with DGF that did and did not require readmission following transplant.

In this study, 59.2% of recipients experienced DGF following transplant. Despite 13.3% of recipients receiving high KDPI kidneys, 29.7% receiving DCD kidneys and 48.4% receiving AKI kidneys, the overall median hospital length of stay was 3 days and the majority of recipients either did not have any readmissions (56.1%) or had an isolated single admission (26.8%). These findings are consistent with our center’s experience in using DGF at-risk allografts (2-3,13). Only a small percentage (17.1%) of recipients with DGF required multiple readmissions. Those recipients were more likely to be diabetic and have longer dialysis vintage. In comparing graft characteristics between those with 0, 1, and ≥2 readmissions high KPDI, DCD and AKI kidneys remained equally represented suggesting that use of DGF at-risk allografts does not necessarily result in increased length of hospital stay and readmissions.

DGF continues to be viewed as an adverse event within the transplant community. This negative connotation associated with DGF is largely driven by studies suggesting a correlation between hospital readmissions, increased resource utilization and other inferior outcomes possibly as a result of surgical complications, infection and rejection (4-5,14-15). These concerns likely limit broader utilization of kidneys at risk for DGF, such as those coming from AKI, high KDPI and DCD donors (8,16). More recent studies have suggested that there is in fact significant heterogeneity within DGF (1-3,17-18). In this cohort, we found that the majority of recipients with DGF had excellent patient and graft survival and lower graft survival was noted only for those with ≥2 readmissions; this finding remained true when accounting for death-censored graft loss (Figure 2) (14-15). This finding was further supported by data coming from mate kidney comparisons. Differences in patient or graft survival were not observed in mate kidneys with and without DGF (Figure 4) or mate kidneys with DGF with and without early readmission (Figure 5). These findings suggest that other factors, independent of DGF, are responsible for kidney transplant outcomes. Despite broad representation of kidney allografts coming from high KDPI, DCD and AKI donor, only a small subset of recipients, those requiring ≥2 admissions, demonstrated inferior survival. For that subset of recipients, comorbidities related to diabetes and dialysis vintage likely played a significant contributing role in outcomes (19-20). Based on this experience, one can conclude that transplant outcome determinants are influenced by the presence and severity of recipient comorbidities, rather than DGF (19-21). Given this risk, additional attention should be given to recipients with early frequent readmissions to try to mitigate longer-term inferior outcomes (20).

Competing variables contribute to transplant outcomes (1,19). As such, active risk reduction strategies should be undertaken for factors that are able to be controlled. In this study, the majority of recipients received depleting induction and had calcineurin inhibitors (CNIs) started early post-transplant despite the presence of DGF. As a result, the overall prevalence of early ACR was notably low. Delay in CNI initiation, along with use of non-depleting induction, in the setting of DGF, has resulted in a body of literature linking DGF, ACR and early allograft fibrosis (22-24). Early initiation of CNIs, with achievement of therapeutic trough levels is, in fact, an important risk modifier that should be undertaken in the setting of DGF (2-3). Similarly, infection, renal recovery related factors and surgical complications accounted for many early readmissions although outcomes were not affected when these events were self-limited (21). For our center, there may be an opportunity to use less depleting induction while still minimizing risk for early rejection through early aggressive initiation of CNIs. Other potential strategies might include improvements in post-transplant diabetes management thereby reducing hyperglycemia and infection risk, as well as a modified outpatient protocol for those presenting with their first hospital readmission. Closer monitoring of patients presenting with their first readmission with a dedicated outpatient care team would perhaps reduce risk for subsequent admissions and adverse outcomes. As such, strategies to minimize recurring readmission events, particularly in the context of recipient comorbidities such as diabetes, warrants further consideration (1,19).

To our knowledge, this is the first study assessing both variables and outcomes specific to DGF kidneys as well as differences in readmission outcomes. It is, however, important to note that, as a single center study, there are limitations as a result of biases introduced through center-specific protocolized practices. As a center with experience in DGF at-risk kidney allograft utilization, the outcomes described here are reflective of carefully selected organs. Donor-recipient pairing remains a crucial component influencing outcomes. Nonetheless, we feel that this data is meaningful. DGF is common event that continues to be associated with a negative connotation throughout the transplant community. More broadly, this association has been linked to kidney allografts that have good outcomes, such as those from DCD and AKI donors, and continues to be a deterrent to broader utilization. As such, we hope to improve utilization of these discard at-risk organs by sharing our experience.

DGF is a common occurrence in high KDPI, DCD and AKI kidneys. Patients with DGF have excellent outcomes as a whole, however, patients with DGF requiring ≥2 readmissions have lower graft survival compared to those with DGF and 0 or 1 readmissions. Independent of DGF, the presence and severity of recipient comorbidities affect transplant outcomes. A better understanding of recipient variables contributing to multiple readmissions may allow for better utilization of DGF at-risk kidneys.

## Data Availability

The raw data supporting the conclusion of this article will be made available by the authors, without undue reservation.

## References

[1] Jadlowiec CC Hanna WA Ninan J Ryan MS Das DM Smith M Transplant Outcomes Using Kidneys from High KDPI Acute Kidney Injury Donors. Clin Transpl (2021) 35(5):e14279. 10.1111/ctr.14279 33690907

[2] Jadlowiec CC Heilman RL Smith ML Khamash HA Huskey JL Harbell J Transplanting Kidneys from Donation after Cardiac Death Donors with Acute Kidney Injury. Am J Transpl (2020) 20(3):864–9. 10.1111/ajt.15653 31612611

[3] Heilman RL Smith ML Kurian SM Huskey J Batra RK Chakkera HA Transplanting Kidneys from Deceased Donors with Severe Acute Kidney Injury. Am J Transpl (2015) 15(8):2143–51. 10.1111/ajt.13260 25808278

[4] Butala NM Reese PP Doshi MD Parikh CR. Is Delayed Graft Function Causally Associated with Long-Term Outcomes after Kidney Transplantation? Instrumental Variable Analysis. Transplantation (2013) 95(8):1008–14. 10.1097/TP.0b013e3182855544 23591726 PMC3629374

[5] Yarlagadda SG Coca SG Formica RN Jr. Poggio ED Parikh CR. Association between Delayed Graft Function and Allograft and Patient Survival: a Systematic Review and Meta-Analysis. Nephrol Dial Transpl (2009) 24(3):1039–47. 10.1093/ndt/gfn667 19103734

[6] Kim DW Tsapepas D King KL Husain SA Corvino FA Dillon A Financial Impact of Delayed Graft Function in Kidney Transplantation. Clin Transpl (2020) 34(10):e14022. 10.1111/ctr.14022 PMC8415124 32573812

[7] Serrano OK Vock DM Chinnakotla S Dunn TB Kandaswamy R Pruett TL The Relationships between Cold Ischemia Time, Kidney Transplant Length of Stay, and Transplant-Related Costs. Transplantation (2019) 103(2):401–11. 10.1097/TP.0000000000002309 29863580

[8] Hart A Lentine KL Smith JM Miller JM Skeans MA Prentice M OPTN/SRTR 2019 Annual Data Report: Kidney. Am J Transpl (2021) 21(2):21–137. 10.1111/ajt.16502 33595191

[9] Doshi MD Garg N Reese PP Parikh CR. Recipient Risk Factors Associated with Delayed Graft Function: a Paired Kidney Analysis. Transplantation (2011) 91(6):666–71. 10.1097/TP.0b013e318209f22b 21317839

[10] Irish WD McCollum DA Tesi RJ Owen AB Brennan DC Bailly JE Nomogram for Predicting the Likelihood of Delayed Graft Function in Adult Cadaveric Renal Transplant Recipients. J Am Soc Nephrol (2003) 14(11):2967–74. 10.1097/01.asn.0000093254.31868.85 14569108

[11] Carey EJ Steidley DE Aqel BA Byrne TJ Mekeel KL Rakala J Six-minute Walk Distance Predicts Mortality in Liver Transplant Candidates. Liver Transpl (2010) 16(12):1373–8. 10.1002/lt.22167 21117246

[12] Heilman RL Smith ML Smith BH Kumar A Srinivasan A Huskey JL Long-term Outcomes Following Kidney Transplantation from Donors with Acute Kidney Injury. Transplantation (2019) 103(9):e263–72. 10.1097/TP.0000000000002792 31205261

[13] Lim WH Johnson DW Teixeira-Pinto A Wong G. Association between Duration of Delayed Graft Function, Acute Rejection, and Allograft Outcome after Deceased Donor Kidney Transplantation. Transplantation (2019) 103(2):412–9. 10.1097/TP.0000000000002275 29762458

[14] Mour G Chang YH Calderon E Chang JM Velazco C Jadlowiec C Kidney Donor Profile index and post-transplant Health Care Utilization: Implications for Value of Transplant Care Delivery. Clin Transpl (2022) 19:e14618. 10.1111/ctr.14618 35182437

[15] King EA Bowring MG Massie AB Kucirka LM McAdams-DeMarco MA Al-Ammary F Mortality and Graft Loss Attributable to Readmission after Kidney Transplantation: Immediate and Long-Term Risk. Transplantation (2017) 101(10):2520–6. 10.1097/TP.0000000000001609 27941434 PMC5462864

[16] McAdams-Demarco MA Grams ME King E Desai NM Segev DL. Sequelae of Early Hospital Readmission after Kidney Transplantation. Am J Transpl (2014) 14(2):397–403. 10.1111/ajt.12563 PMC3998748 24447652

[17] Heilman RL Mathur A Smith ML Kaplan B Reddy KS. Increasing the Use of Kidneys from Unconventional and High-Risk Deceased Donors. Am J Transpl (2016) 16(11):3086–92. 10.1111/ajt.13867 27172238

[18] Budhiraja P Reddy KS Butterfield RJ Jadlowiec CC Moss AA Khamash HA Duration of Delayed Graft Function and its Impact on Graft Outcomes in Deceased Donor Kidney Transplantation. BMC Nephrol (2022) 23(1):154. 10.1186/s12882-022-02777-9 35440023 PMC9017045

[19] Phillips BL Ibrahim M Greenhall GHB Mumford L Dorling A Callaghan CJ. Effect of Delayed Graft Function on Longer-Term Outcomes after Kidney Transplantation from Donation after Circulatory Death Donors in the United Kingdom: A National Cohort Study. Am J Transpl (2021) 21:3346–55. 10.1111/ajt.16574 33756062

[20] Matas AJ Gillingham KJ Humar A Ibrahim HN Payne WD Gruessner RW Posttransplant Diabetes Mellitus and Acute Rejection: Impact on Kidney Transplant Outcome. Transplantation (2008) 85(3):338–43. 10.1097/TP.0b013e318160ee42 18301329

[21] Meier-Kriesche HU Kaplan B. Waiting Time on Dialysis as the Strongest Modifiable Risk Factor for Renal Transplant Outcomes: a Paired Donor Kidney Analysis. Transplantation (2002) 2774(10):1377–81. 10.1097/00007890-200211270-00005 12451234

[22] Luan FL Barrantes F Roth RS Samaniego M. Early Hospital Readmissions post-kidney Transplantation Are Associated with Inferior Clinical Outcomes. Clin Transpl (2014) 28(4):487–93. 10.1111/ctr.12347 24579998

[23] Wu WK Famure O Li Y Kim AJ. Delayed Graft Function and the Risk of Acute Rejection in the Modern Era of Kidney Transplantation. Kidney Int (2015) 88:851–8. 10.1038/ki.2015.190 26108067

[24] Nankivell BJ Kuypers DR Fenton-Lee CA Allen RD O'Connell PJ Chapman JR. Histological Injury and Renal Transplant Outcome: the Cumulative Damage Hypothesis. Transpl Proc (2001) 33(1-2):1149–50. 10.1016/s0041-1345(00)02806-2 11267231

