# Supplementary material for: Association of DGF and Early Readmissions on Outcomes Following Kidney Transplantation
Source: Transpl Int. 2022 Dec 23;35:10849. doi: 10.3389/ti.2022.10849 (PMC9817097; doi:10.3389/ti.2022.10849)
Supplement: Supplementary file 1 [file Table1.pdf]

Table S1 – Cox Regression Model

	**Univariate**			**Multivariate**		
	HR	95% CI	*P* value	HR	95% CI	*P* value
≥2 Readmissions	3.25	1.90-5.58	<0.001	3.12	1.81-5.37	<0.001
Diabetes	1.41	0.83-2.40	0.20	1.28	0.75-2.19	0.36
Dialysis Vintage	1.06	0.99-1.14	0.11	1.06	0.98-1.14	0.14
