# Supplementary material for: Association of DGF and Early Readmissions on Outcomes Following Kidney Transplantation
Source: Transpl Int. 2022 Dec 23;35:10849. doi: 10.3389/ti.2022.10849 (PMC9817097; doi:10.3389/ti.2022.10849)
Supplement: Supplementary file 2 [file DataSheet1.pdf]

| | **Missing data** (Total=922) |
|---|---|
| **Recipient** | |
| - Age | 0 (0.0%) |
| - Sex | 0 (0.0%) |
| - Race | 0 (0.0%) |
| - Diabetes | 0 (0.0%) |
| - Ejection Fraction | 34 (3.7%) |
| - 6-minute walk distance | 0 (0.0%) |
| - Midodrine | 0 (0.0%) |
| - Dialysis/Preemptive | 6 (0.7%) |
| - Length of dialysis | 23 (2.5%) |
| - Re-transplant | 0 (0.0%) |
| - Induction | 0 (0.0%) |
| **Donor** | |
| - Age | 0 (0.0%) |
| - Sex | 0 (0.0%) |
| - Height | 42 (4.6%) |
| - KDPI | 0 (0.0%) |
| - DCD | 0 (0.0%) |
| - AKI | 70 (7.6%) |
| - Allocation | 0 (0.0%) |
| - CIT | 0 (0.0%) |
| - LOS | 12 (1.3%) |
| - DGF days | 96 (10.4%) |
| - ACR events | 0 (0.0%) |
| - eGFR | 97 (10.5%) |
